# Accidental Release of Chlorine from a Storage Facility and an On-Site Emergency Mock Drill: A Case Study

**DOI:** 10.1155/2015/483216

**Published:** 2015-06-10

**Authors:** Ambalathumpara Raman Soman, Gopalswamy Sundararaj

**Affiliations:** ^1^Department of Mechanical Engineering, Government Engineering College, Thrissur, Kerala 680 009, India; ^2^Department of Mechanical Engineering, PSG College of Technology, Coimbatore, Tamil Nadu 641 004, India

## Abstract

In the current industrial scenario there is a serious need for formulating strategies to handle hazardous substances in the safest way. Manufacture, storage, and use of hazardous substances pose a serious risk to industry, people, and the environment. Accidental release of toxic chemicals can lead to emergencies. An emergency response plan (ERP) is inevitable to minimize the adverse effects of such releases. The on-site emergency plan is an integral component of any process safety and risk management system. This paper deals with an on-site emergency response plan for a chlorine manufacturing industry. It was developed on the basis of a previous study on chlorine release and a full scale mock drill has been conducted for testing the plan. Results indicated that properly trained personnel can effectively handle each level of incidents occurring in the process plant. As an extensive guideline to the district level government authorities for off-site emergency planning, risk zone has also been estimated with reference to a chlorine exposure threshold of 3 ppm.

## 1. Introduction

From time and again the ever increasing and complex processing of hazardous chemicals in chemical process industries is a potential danger to the public and environment. Accidents in process industry may occur due to failure of process equipment, operational mistakes, and human error or due to external interruptions like natural disasters. Different countries have their own federal regulations to control the risks in the operation of hazardous facilities, and some of it are Occupational Safety and Health Administration's (OSHA) Process Safety Management (PSM) standard and the Environmental Protection Agency's (EPA) Risk Management Program (RMP), and Manufacture, Storage, and Import of Hazardous Chemicals Rules (MSIHC). Though considerable efforts have been made to prevent industrial accidents, still accidents are being repeated in chemical process industries [1–3]. Thus, in addition to the preventive measures, specific emergency management measures are also required to reduce the consequences of accidents associated with industrial hazards. Preventive measures are the most significant approach to progress the safety in industrial activities, at the same time the emergency management measures are also significant. Emergency management refers to the deployment of science, technology, planning, and management to deal with extreme events, damage to property and community life [4]. Therefore, the emergency authorities concerned are required to prepare themselves and be in a state of readiness at all times to tackle any emergency situations. To attain this state of readiness, systematic training should be given to the personnel involved in the emergency response plan (ERP). Development of well-defined and suitable ERP requires a systematic review of hazards associated with plant facility and their consequences. The worst-case scenarios are generally used in the consequence assessment for analyzing the most severe hazard that might occur in a process plant [5]. During the recent years, researchers have been appraising the power of computer aid consequence model for the real time evaluation of an emergency. There are number of consequence-modeling software available such as SLAB, PHAST, SAFETI, DEGADIS, BREEZE HAZARD, and HYSIS [6–10] which are finding a growing acceptance in the preparation of ERP. Various studies have been reported that an effective ERP is necessary for process industry in order to reduce the consequences of accidents associated with industrial activities [11–14]. Though a lot of authors have reported that mock drills are unavoidable for ERP to test the effectiveness, only few authors have explained the sequences of action taken during the mock drill which is very essential to understand the various safety measures for incorporation into future research. Well-rehearsed and widely understood and accepted planning procedures are the key factors in the ERP to protect human life, property, and environment. It has been noticed that the on-site emergency plan is being made with discrete elements and there is a need to develop a uniform structured on-site ERP for the case study plant considered.

This paper illustrates an attempt to develop a structured and pertinent emergency response plan for a chlorine storage facility and implementing it. A case study based approach has been used for conducting a mock drill and subsequent analysis.

## 2. Methods

### 2.1. Chlorine Storage Facility

**Figure 1:**
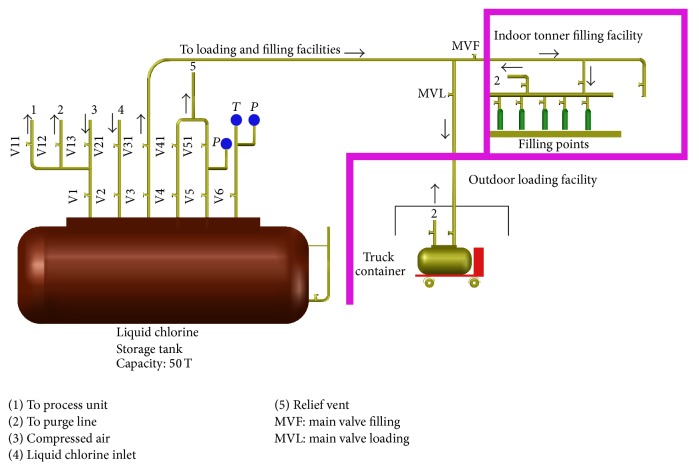
Chlorine storage, tonner, and truck container loading facility.

The major products of chlor-alkali industry are chlorine, hydrogen, and sodium hydroxide (caustic) solution. Of these, the primary product is chlorine. Chlorine is liquefied and stored in a horizontal cylindrical tank of capacity 50 T. The storage tank is provided with twin relief vent safety system, rupture disc, level indicator, pressure gauge, safety valve inlet, and outlet connections. A partial pressure of 3 kg/cm^2^ and temperatures of −10°C are maintained in the storage tank. The diameter and length of the horizontal cylindrical dished end tank are 250 mm and 8830 mm, respectively. The chlorine storage facility is provided with indoor tonner loading facility and outdoor truck container loading facility. The compressed dry air at 10 kg/cm^2^ is used for transferring the liquid chlorine from storage facility to truck container or to tonner filling facility. The chlorine truck container is parked in outdoor loading area and is provided with the connections for filling and purging.  Figure 1 shows the details of chlorine storage tank, tonner filling, and truck container loading facility.

### 2.2. Chlorine Release and Coverage Zone

Chlorine is a greenish-yellow gas with a pungent and irritating odor and it finds wide applications in chemical industries. It is an acute poisonous dense gas and can be inhaled into human bodies through respiratory systems. Chlorine gas is extremely irritating to the mucous membranes, the eyes, the respiratory tract, and spine. This heavy gas can accumulate at ground level, so that an unexpected release exceeding its threshold value might ultimately cause human injury or death [15]. Its occupational exposure threshold is 1 mg/m^3^ and a small amount of chlorine can quickly reach the threshold of acute poisoning and cause casualties [16].  Table 1 shows the physical properties of chlorine [17].

Various studies on the release of chlorine from the storage facility have been reported [18–20].  Table 2 shows chlorine exposure thresholds and reported responses in humans [21].

In this paper, a previous study on chlorine release scenario on the basis of engineering judgment [22] was considered for the preparation of emergency response plan. A mock scenario of a chlorine discharge occurring from a 16 mm diameter pipe connected to the base of the storage tank for sight glass fitting was considered. The quantity of chlorine in the tank was 45.0 T which was maintained at a pressure of 3 kg/cm^2^ and the corresponding height of liquid was 1.55 meters. The discharge rate calculations have been performed for the release of chlorine in liquid-gas phase.

The discharge rate of chlorine in liquid-gas phase is given by [23]

(1) $$\begin{array}{c} Q=AC_{d}f\left(\;l\;\right)\sqrt{P_{1}}, \end{array}$$

where *Q* is the discharge rate (kg/s), *A* is the cross sectional area of pipe (m^2^), *C*_*d*_ is the coefficient of discharge (0.62), *f*(*l*) is the friction factor (22.36) [23], and *P*_1_ is the upstream pressure in the storage tank (N/m^2^). The dispersion calculation provides an estimate of the area affected and the average vapor concentration at the ground level. The ground level concentration of chlorine along the centerline is given by [24]

(2) $$\begin{array}{c} C_{G}=\frac{Q}{\pi u\sigma_{y}\sigma_{z}}, \end{array}$$

where *C*_*G*_ is the ground level concentration of chlorine (kg/m^3^), *u* is the wind velocity (3 m/s), and *σ*_*y*_ and *σ*_*z*_ are the horizontal and vertical dispersion coefficients (m), respectively. The dispersion coefficients are calculated using the downwind distance and stability category of the atmosphere. The stability categories are designated as A to F which provides the degree of vertical mixing in the atmosphere. The stability category F in urban condition is considered for this study.

The vertical dispersion coefficient is given by

(3) $$\begin{array}{c} \sigma_{y}=0.11x{\left(\;1+0.0004x\;\right)}^{-1/2}. \end{array}$$

**Figure 2:**
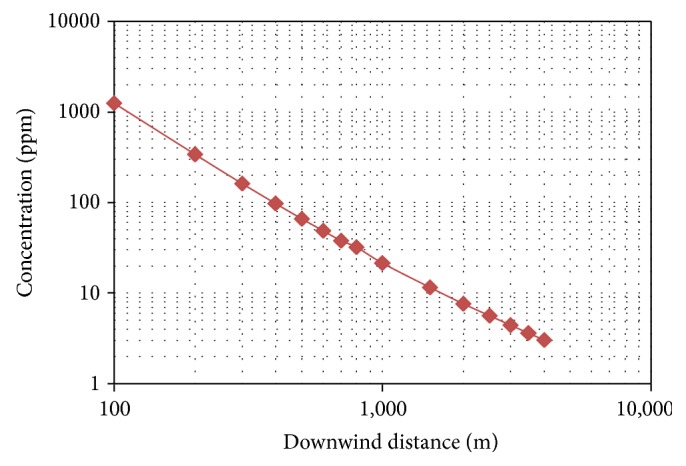
Downwind distance of chlorine release (liquid-gas phase).

**Figure 3:**
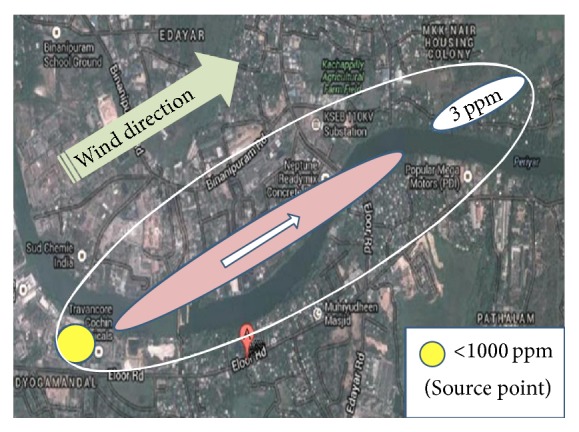
Risk area and coverage zones for chlorine release (source: Google map).

The horizontal dispersion coefficient is given by

(4) $$\begin{array}{c} \sigma_{z}=0.08x{\left(\;1+0.0015x\;\right)}^{-1/2}, \end{array}$$

where *x* is the downwind distance (m). The dispersion model indicates that the release of chlorine is likely to result in threshold limit value of 3 ppm at a downwind distance of 4 km as shown in Figure 2. The risk area and coverage zone for chlorine release scenario are shown in Figure 3.

### 2.3. Emergency Response Plan

**Figure 4:**
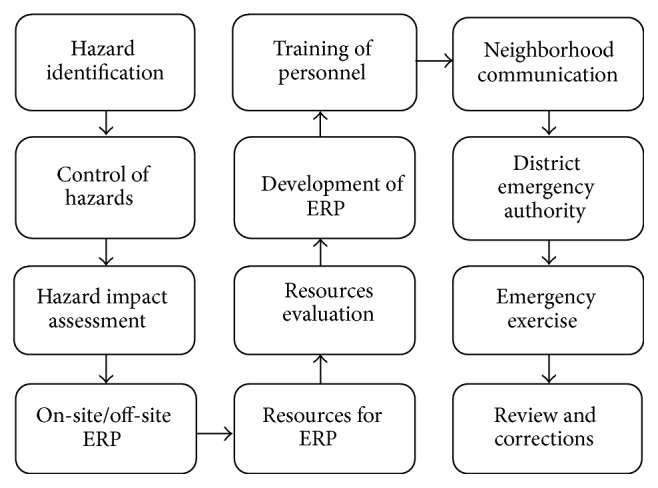
Outline of emergency response plan.

The major component of emergency preparedness is a formal written emergency plan developed on the basis of identified probable accidents together with their consequences [25]. The key consideration of the plan is protection of the people, property, and the environment from harm during an emergency situation. It also focuses on a range of time-sensitive tasks that need to be undertaken involving efforts at all levels, minimizing the effects of the incident on personnel, property, and environment.  Figure 4 shows the outline of the emergency response plan which is based on the hazard identification, prediction, and prevention. Hazard identification and evaluation study have to be carried out prior to creation of ERP to identify potential hazardous situations that could occur in an industry and to find out possible control measures.

Several methods like check list procedure, what if analysis, concept hazard analysis, failure mode and effects analysis, and hazard and operability (HAZOP) study are available for the identification and evaluation of hazards in the chemical process industries. Of these, more creative and open-ended procedure is HAZOP study [26, 27]. A detailed review on HAZOP analysis has been reported by Dunjó et al. [28]. Recently expert systems based computer aided software has been developed for hazard identification which resulted in significant reduction in manpower resources [29–32].

The ERP must contain the following activities:

- identification of hazardous chemicals, processes, and the facilities,
- release scenarios and discharge rate measurement,
- consequences in terms of toxic concentration,
- dispersion analysis for toxic release,
- preparation of site plan for damage control,
- identification of the vulnerable zones,
- classification of process unit or units which have the most potential for creating on-site and off-site emergency plans,
- identification of the facilities available in the vulnerable zone,
- identification of the requirements of various departments in the process plant for coping with emergency situation.

#### 2.3.1. Emergency Response Organization

At the outset, for the development of an ERP, all the participants and departments should be identified and their roles, resources, activities, and capabilities should be established.

All the members in the organization should be trained to tackle the emergency effectively. The factors considered for the development of emergency response organization are

- identification of resource person as emergency controller at emergency control center (ECC),
- identification of resource person as incident controller at site,
- allocation of resources to mitigate the consequences of chlorine release,
- resource person responsible for effective communication,
- types of protective action like evacuation or shelter,
- earlier reports of handling emergency situations,
- consequence assessment reports,
- responsible person for recovery action.

Considering the above factors, an emergency response organization, complete with command structure were formed. The Assistant General Manager-Operation [AGM (O)] in charge of production cum plant operation will act as emergency controller (EC). AGM (O) will take overall control of the emergency due to chlorine release and he will be in charge of ECC. The Deputy Manager-Shift Duty [DM (SD)] will act as incident controller (IC). He will take emergency control on scene in the event of chlorine release.

#### 2.3.2. On-Site Emergency Plan

Emergency planning is pursued on-site and off-site. An off-site emergency plan involves a lot of external variables and is very difficult to organize. Therefore, onsite emergency planning is considered for this research. If an incident takes place inside a factory and its effects are confined to within the factory premises, involving only the persons working in the factory and the properties inside the factory, it is called an on-site emergency. An on-site emergency plan needs to contain a scale plan of the plant along with the list of hazardous chemicals handled, indicating the quantities involved and their locations relative to the surrounding area and population.

#### 2.3.3. Resources

Identification of the resources available to the implementation of ERP is an essential part of the planning process. To implement any emergency response plan, the appropriate personnel, facilities, equipment, and supplies must be available. Some of the resources could already be available in the industry. A list of those items unavailable for the ERP was prepared and presented to the plant management. It is the responsibility of plant management to ensure that the appropriate equipment and supplies to tackle their emergencies are available at the plant. The resources appropriate for their anticipated response activities were identified.

#### 2.3.4. Emergency Control Centre

**Figure 5:**
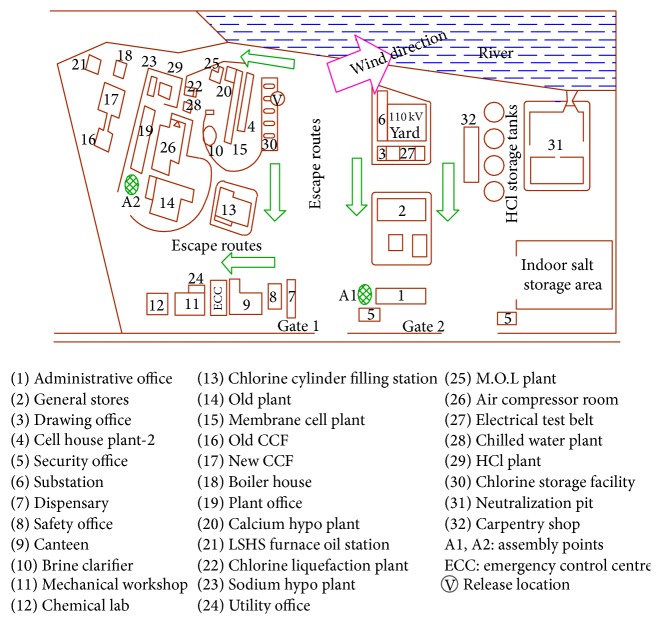
Industry map showing the escapes routes, assembly points, and emergency control centre.

All chemical process industries should establish an emergency control center ECC from which response activities can be directed and coordinated whenever a major emergency is declared. The ECC should be located where the risk of exposure to accidental release is minimal and should be close to the routes in that the personnel can reach it easily.  Figure 5 shows the map of the industry indicating ECC, escape routes, and assembly points.

Upon emergency the ECC will be activated by a person in charge of operation. The ECC should always be ready or set up quickly for operation. The ECC should contain the following:

- up to date copies of the ERP and implementation procedures,
- emergency telephone registers,
- details of external agencies, response organization, and neighboring industries,
- adequate communication facilities like mobile phone and land telephone (some outgoing only) for communicating with internal and external agencies,
- fax machine,
- Internet connected computer system with dispersion model software,
- material safety data sheets (MSDS) for the chemicals at the site,
- maps of the plant and surrounding areas,
- latest report of consequence assessment of chlorine release,
- emergency power supply,
- knowledge about the types of personal protective equipment and its locations,
- lay out maps to indicate
  - area(s) affected by the accident,
  - position and movement of the vapour clouds,
  - position of the response teams,
  - evacuation areas and assembly points.

The office of the DM (SD) was identified as emergency control center for the present emergency response plan.

#### 2.3.5. Communication

**Figure 6:**
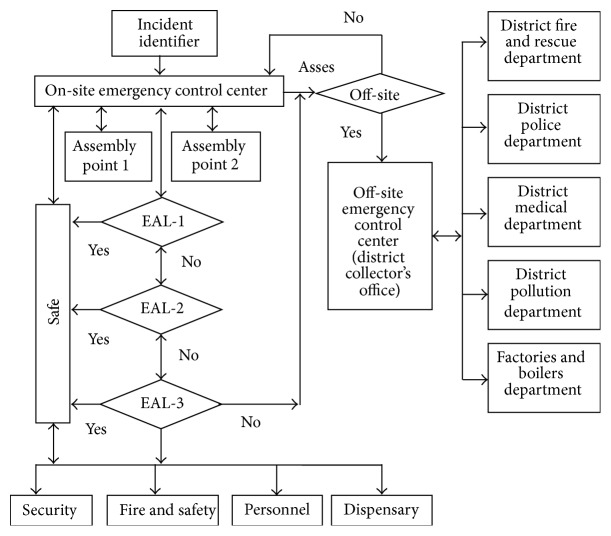
Network communication systems for emergency response plan for chlorine release.

The importance of communication network in emergency planning lies in its ability to get people to work together on a common task. Coordination of task accomplishment and resource management between different departments requires efficient and effective interagency communication. A communication network for the proposed emergency plan was prepared and presented in Figure 6. It should ensure that the flow of communications within the onsite response organization and between the organization and off-site response organizations and agencies is effective and uninterrupted. Network communication occurs between EAL-1, EAL-2, and EAL-3. The lowest Emergency Action Level (EAL-1) may be associated with chlorine release that is either under control or can be easily brought under control by plant personnel/incident identifier in the immediate area. This intermediate emergency level (EAL-2) is associated with chlorine releases that affect more than the immediate area but have not spread beyond the plant boundary. EAL-3 implies that the accident has the potential for beyond the plant boundaries. If EAL-1 cannot control the situation, it is communicated to EAL-2 and so on.

#### 2.3.6. Roles and Responsibilities

The roles and responsibilities of facility response teams from the plant emergency organization were identified and presented. Release of chlorine from the storage facility is detected by the sensor and the alarm. Once the alarm is accessed the responsibilities of the key personnel are narrated herewith. The shift in charge at plant site [SIC-1] is considered as the first person responds to the alarm.

*Responsibilities of [SIC-1]*. On receiving the alarm indication SIC-1 should

- instruct the charge man at plant site [CM-1/CM-2] to ensure the ammonia torch and breathing apparatus are ready to inspect chlorine release,
- inspect the correct location of the release,
- examine the state of release, whether release is in liquid or gaseous form,
- recognize the leak location and nature of leak,
- put up actions for remedial measures, if any,
- inform the shift in charge at process control room SIC-2 regarding the chlorine release scenario,
- sound like the hand siren,
- check the wind direction and watch the surrounding.

*Responsibilities of Shift in Charge at Process Control Room [SIC-2]*. On receiving the information from anyone noticing the chlorine release, SIC-2 should

- inform the Senior Engineer-Operation SE (O) and Deputy Manger-Operation DM (O), either by loudspeaker announcement or by telephone,
- alert the shift in charge of concerned plants such as chlorine filling station, air compressor station, chlorine liquefaction plant, hydrochloric acid plant, waste chlorine disposal plant, sodium hypochlorite plant, dispensary, and security,
- awaiting information from anyone for further actions.

*Responsibilities of Deputy Manager-Operation [DM (O)]*. On receiving the information from SIC-2, DM (O) should

- visit the release area and assess the emergency situation,
- instruct SE (O) to check the level of chlorine in the storage and ensure one empty storage tank should be available for chlorine transferring,
- instruct SIC-2 to inform Plant Manager-Operation (PLM) (O), incident controller (IC), Chief Engineer-Maintenance (CE (M)), and Chief Engineer-Utility CE (U) through loud speaker or telephone,
- ask safety and rescue department to send the additional breathing apparatus,
- check the wind direction and watch the area.

*Responsibilities of Incident Controller [DM (SD)]*. On receiving the information from DM (O) through loud speaker announcement by SIC-1, the DM (SD) should

- visit the release location and assess the emergency situation,
- alert the Emergency Controller AGM (O) through mobile phone,
- ask safety personnel to bring adequate personnel protective equipment,
- instruct the SE (O) to isolate the tank to stop chlorine flow into the tank,
- inform the CE (M) to coordinate the mitigation actions with abatement crew,
- take steps to transfer chlorine from the leaked tank to empty storage tank,
- activate water curtain to minimize the chlorine dispersion into surrounding,
- take steps to add caustic flakes to leaked area for neutralization of leaked chlorine,
- call security personnel to control the vehicle movement and personnel intervention at the site,
- check the wind direction and instruct the SIC-2 to announce the probable hazardous area so that anyone can take safe actions accordingly,
- take step to stop all regular job activities at overhead site,
- obtain any help from external agency through AGM (O),
- document the emergency actions completed and review the actions,
- inform the AGM (O) by cellular phone regarding the present and anticipated condition.

*Responsibilities of Emergency Controller [AGM (O)]*. On receiving the alert from incident controller DM (SD), the AGM (O) takes charge of Emergency Control Center (ECC). The AGM (O) should

- review the information and confirm the release scenario with earlier consequence assessment reports,
- assess the weather condition and wind velocity using Internet,
- alert the District Collector about the nature of the release, expected area and population that get affected, remedial measures taken, and so forth,
- inform the nearby company authorities, pollution control board, police, and medical service,
- arrange additional help to DM (SD) from external/internal departments,
- inform Chief Security-Officer CS(O) to clear the all passageway and control the vehicles into the company,
- inform the Chief Engineer-Safety CE (S) to coordinate on-site safety activities at site,
- inform Medical Officer MO to ensure all medical facilities should be available,
- inform the personnel department about the nature of the accident to ensure all facilities including transportation, communication with relatives of affected people, and so forth.

*Responsibilities of Chief Engineer-Safety [CE (S)]*. On receiving information from AGM (O), the CE (S) should

- review the information from AGM (O),
- alert the safety officers in the safety department,
- visit the site where release has occurred,
- ensure the availability of personal protective equipment like breathing, apparatus, goggles, helmet, gloves, acid alkali resistant jacket, canister and cartridge type mask, and so forth,
- coordinate on-site safety activities including first aid and evacuation.

*Responsibilities of Medical Officer [MO]*. On receiving information from AGM (O), MO should

- review the information from AGM (O),
- alert all supporting staff in the medical department,
- communicate with AGM (O) through mobile phone for additional help,
- arrange first aid/hospitalization of affected people,
- keep the record that includes the nature of injury, type of treatment given, and type of treatment required for affected peoples,
- identify and inform the hospital nearby for further emergency treatment.

*Responsibilities of Chief Security Officer [CS (O)]*. On receiving information from AGM (O), the CS (O) should

- review the information from AGM (O),
- alert all the security officers in the security department,
- communicate with the AGM (O) through radio or mobile phone,
- sound like the siren for emergency,
- control the traffic of vehicles and clear the escape route,
- assist safety department in evacuation of injured persons.

*Responsibilities of Personnel Manager [PM]*. On receiving information from EC, the PM should

- review the information from AGM (O),
- alert concerned staff in the personnel department,
- communicates with AGM (O) through phone,
- collect and record the details of affected persons and inform their relatives,
- arrange transport facility whenever required,
- ensure the ambulance is ready for emergency,
- ensure injured peoples are getting sufficient attention and required treatment facility.

### 2.4. Implementation of Emergency Response Plan

A set of procedure for the implementation of on-site emergency response plan as described above has been developed and circulated to all concerned departments in the case study plant. After the procedure is prepared, the ERP, the way it was implemented, and its effectiveness in bringing the emergency under control should be reviewed through mock drills. Prior to conducting the mock drill, detailed training was given to all persons in the emergency response organization to do the activity they have been assigned.

### 2.5. Training Program

Training was provided to all personnel in the emergency response organization and their evaluation and inferences incorporated so that they are familiar with their roles and responsibilities in the event of an emergency. Areas covered in the training programs include

- general duties, roles, and responsibilities under the plan,
- emergency functions of the organizational structure,
- emergency procedures,
- emergency resources.

Specific training programs for personal were conducted at the chlorine storage facility with the help of human resource department in the industry concerned.

### 2.6. Mock Drill

The mock drills and exercises are vital to emergency response plan as it is a performance test for the resources during an emergency. So an emergency response plan should be accompanied by a mock drill/exercise. The concerned authority should ensure that a mock drill of the on-site emergency response plan is conducted every six months. The objectives of the mock drills are [33]

- to familiarize personnel with content of the plan and its manner of implementation,
- to train new personnel or personnel who move within the plant organization,
- to train specific response personnel in particular duties requiring special skills,
- to introduce personnel to new equipment, techniques, and concept of operation,
- to keep personnel informed of changes in the plan or procedures,
- to test the preparedness of response personnel,
- to test the validity, effectiveness, timing, and content of the plan and implementing procedures,
- to test emergency equipment,
- to update and modify the plan on the basis of the experience acquired through mock drills,
- to maintain cooperative capability with local response departments, organizations, and jurisdictional agencies,
- to maintain good emergency response capability.

Prior to conducting the full scale mock drill, the onsite emergency plan developed was tested using tabletop models. Tabletop drills are useful for orientation purposes to the concerned personnel using scale models and the emergency situation put forward which has to be resolved. The deficiencies in the onsite emergency plan were identified and corrected accordingly.

### 2.7. Sequences of Actions Taken

A hypothetical accident scenario of chlorine release as mentioned earlier was used in the testing of the on-site emergency plan. The population within the vulnerable area was 60. The observers for mock drill were identified well in advance. The Chemical Inspector from factories and boilers department was presented as observer from government nominee. All observers participating in the mock drill were briefed prior to the full scale mock drill and provided with a check list for recording the actions during the drill. The full scale mock drill was initiated by alerting shift in charge of control room through alarm at 10.30 am that an emergency has occurred in chlorine storage area. On receiving the information through alarm the mock drill was imitated and the drill sequences are as follows.

 10.30 am: an emergency was detected by SIC-2 through local alarm at the control room. Information passed from SIC-2 to the SIC-1 at the chlorine storage facility.
 10.31 am: SIC-1 instructs CM-1 to inspect chlorine release with ammonia torch. SIC-1 checks the wind direction and surroundings.
 10.32 am: CM-1 informs SIC-1 that the leak is from number 5 storage tank. He finds CM-2 coughing having inhaled chlorine. CM-1 immediately takes him to safe place; meanwhile, SIC-1 calls dispensary for arranging ambulance. SIC-1 reaches near the victim CM-2.
 10.33 am: CM-1 again goes to check the leak wearing breathing apparatus set. He finds out that the leak is from the pipe leading to the sight glass of number 5 storage tank. CM-1 informs these details to SIC-1.
 10.34 am: SIC-1 asks control room instructing to inform SE (O) and SE (U). The details of chlorine leak and wind direction are also intimated to them. The hand siren is sounded.
 10.35 am: SIC-1 calls safety department to send breathing apparatus/security section to send security persons and control vehicles towards storage area/filling area.
 10.36 am: SE (O) and SE (U) reach storage area and discusses the situation with SIC. SE (O) asks control room to announce chlorine leak and the prevailing wind direction and requests the DM (O) and PL (M) to reach the site. SE (O) calls shift in charge at liquefaction plant and instructs to stop chlorine transfer. Meanwhile, SE (U) calls the abatement crew.
 10.37 am: security personnel reached the site. Meanwhile, ambulance arrives and the victim is evacuated.
 10.38 am: safety personnel arrives at site.
 10.39 am: abatement crew reaches the site. Safety personnel help them to wear BA set.
 10.39 am: personnel received call from liquefaction plant telling they have closed the chlorine transfer line. SE (O) instructs to transfer the chlorine to the empty tank and to purge the line after one minute.
 10.43 am: DM (O) and PL (M) reach the site and discusses the situation with SE (O).
 10.43 am: SE (U) asks the abatement crew to check the leak and try to arrest it.
 10.45 am: PL (M) calls AGM (O), DM (SD), and CE (U) and informs them about the situation.
 10.46 am: SE (O) calls control room and asks to made announcement through the public address system about chlorine leak.
 10.49 am: AGM (O), DM (SD), and CE (U) reach the site and discuss the situation with the PL (M). Meanwhile, abatement crew returns and reports that the leak can be arrested by clamping.
 10.50 am: AGM (O) and DM (SD) analyze the situation and decide to declare an emergency. AGM (O) declares on-site emergency and DM (SD) office is declared as emergency control centre. AGM (O) goes to emergency control centre with PL (M) and on the way he informs General Manager-Technical [GM (T)], AGM (HR) (communication coordinator).
 10.50 am: DM (SD) instructs the abatement crew to clamp the leak and instructs the SIC-1 to activate the water curtain.
 10.50 am: SE (U) arranges a standby abatement crew.
 10.52 am: from emergency control room AGM (O) directs CS (O) to sound the emergency siren, instructs transport coordinator to control vehicle traffic in the plant, and asks control room to caution the plant personnel and ask them to proceed to the assembly points.
 10.52 am: following the direction from AGM (O), CS (O) instructs the security personnel to sound the emergency siren to alert the public and also to keep all the gates open and block all vehicles and personnel entering the premises.
 10.54 am: AGM (O) calls DM (SD) and discusses the progress of work and requests to ensure there is sufficient quantity of caustic soda in soda bleach plant for neutralization.
 10.59 am: abatement crew returns and informs DM (SD) that they have arrested the leak successfully by clamping the line.
 11.00 am: DM (SD) reported the actions taken to AGM (O).
 11.03 am: DM (SD) along with CE (M) inspects the location and advices AGM (O) to lift the emergency. AGM (O) directs CS (O) to sound the all clear sirens and informs the matter to control room and all the other concerned.
 11.04 am: there is announcement from control room that the emergency is lifted.
 11.04 am: security personnel takes the head count and ensures nobody is missing. AGM (O) enquires at dispensary about the injured employees.
 11.05 am: there is joint inspection by the concerned members.

### 2.8. Observations and Recommendations

Immediately after the drill was over, concerned departments and the observers assembled at the room at the AGM (O) for debriefing. The shortcomings and inadequacies were discussed and arrived at a few recommendations given below.

- An adequate number of self-contained breathing apparatus should be provided in the plant. It should be ensured that all working groups in the plant should be trained in using breathing apparatus.
- All drivers of the ambulance should be familiar with plot plan and road maps of the company.
- Security department was asked to be more alert in traffic control at the escape routes and assembly points. They have to ensure that there are no obstructions at those places.
- Civil department was asked to improve housekeeping at chlorine storage areas. It should be ensured that no scrap pipes are dumped in the dyke of chlorine storage.
- Additional wind flag should be installed at the chlorine storage area and assembly points.
- All the concerned staff should be trained in handling people during emergency.
- All the staff should be trained in reading the plot plan which shown the escape routes, road maps, and assembly points.
- One chlorine storage tank should be kept empty and the equipment number of the tank be recorded so that transfer of chlorine can be done in an emergency without delay.
- Proper protective clothing covering the entire body should be provided to the plant employees.
- A strict maintenance regulation should be customized to improve the effectiveness of mitigation measures.
- Safety department was asked to develop a new mitigation system for chlorine storage facility aiming to reduce consequences of chlorine release.

### 2.9. Review and Revision

A detailed report of the mock drill was prepared based on the observations and recommendations made in the debriefing and it was circulated to concerned departments and observers. The on-site emergency plan was revised based on the suggestions received from various departments and observers. The updated versions were issued to all concerned departments for documentation. This procedure has to be continued in the subsequent operations.

## 3. Conclusions

Hazards are always catastrophic threads to any organizations. They always necessitate proper attention to management and mitigation. Emergency response plans visualize measures to contain and minimize the effects due to spillage of hazardous chemicals, accidental release of toxic gases, and fire or explosion. All hazardous installations should have an adequate emergency plan which is applicable to that installation and is based on a complete range of credible accident scenarios. A systematic analysis of hazard identification procedures and consequence analysis is necessary to tackle such situations. This will help to successfully handle major accident scenarios. Key areas of emergency preparedness are good communication system, training in emergency preparedness, education and safety training of the employees, review of past accidents, consequence assessment reports, and regular interaction between various departments. The emergency plan and procedure must be periodically reviewed and updated based on the observations made during full scale mock drills. This is to ensure that they are current and valid, conform to new regulations, and incorporate lessons learnt, and modifications are made to improve effectiveness. In this regard, a systematic mock drill is illustrated in this paper. This research paper may act as a guide for practitioner for successfully handling such accident scenarios. Further, this may help future researchers to model hazards and suggest a safe operating scheme. This study can be beneficial to understand the significant elements for incorporation into off-site emergency management plan. This plan can serve as a handy reference to various response agencies.

## Figures and Tables

**Table 1:** Physical properties of chlorine.

Atomic weight	35.5
Molecular weight	70.905
Boiling point (at 1 atm)	−34.6°C
Melting point	−100.98°C
Viscosity (20°C)	0.346 m Pa s
Color	Yellow-green
Critical temperature	144°C
Critical pressure	76.1 atm
Degree of dissolve of water	0.7 g/100 g H_2_O (20°C)
Specific gravity	2.5

**Table 2:** Chlorine exposure thresholds and reported responses in humans.

Exposure level (ppm)	Effect
0.2–0.4	Threshold of odor perception with considerable variation among subjects
30	Immediate chest pain, vomiting, dyspnea, and cough
430	Lethal over 30 minutes
1,000	Fatal with a few minutes
